# Neuroprotective Effects of Celastrol on Transient Global Cerebral Ischemia Rats via Regulating HMGB1/NF-κB Signaling Pathway

**DOI:** 10.3389/fnins.2020.00847

**Published:** 2020-08-11

**Authors:** Bo Zhang, Qi Zhong, Xuhui Chen, Xi Wu, Rong Sha, Guizhi Song, Chuanhan Zhang, Xiangdong Chen

**Affiliations:** ^1^Department of Anesthesiology, Union Hospital, Tongji Medical College, Huazhong University of Science and Technology, Wuhan, China; ^2^Department of Anesthesiology, Tongji Hospital, Tongji Medical College, Huazhong University of Science and Technology, Wuhan, China; ^3^Department of Anesthesiology, Zhongnan Hospital, Wuhan University, Wuhan, China; ^4^Department of Ophthalmology, Tongji Hospital, Tongji Medical College, Huazhong University of Science and Technology, Wuhan, China; ^5^Department of Rehabilitation Medicine, Enshi Autonomous Prefecture, Hospital of Traditional Chinese Medicine, Enshi, China; ^6^Department of Quality Inspection, Wuhan Institute of Biological Products, Wuhan, China

**Keywords:** celastrol, neuroinflammation, oxidative stress, neurological deficit, cerebral ischemia reperfusion

## Abstract

Cerebral ischemia is a major cause of brain dysfunction, neuroinflammation and oxidative stress have been implicated in the pathophysiological process of cerebral ischemia/reperfusion injury. Celastrol is a potent inhibitor of inflammation and oxidative stress that has little toxicity. The present study was designed to evaluate whether celastrol has neuroprotective effects through anti-inflammatory and antioxidant actions, and to elucidate the possible involved mechanisms in transient global cerebral ischemia reperfusion (tGCI/R) rats. Celastrol (1, 2, or 4 mg/kg) was administrated intraperitoneally immediately after reperfusion and the effect of celastrol on reverting spatial learning and memory impairment was determined by Morris water maze (MWM) task. Inflammatory response and oxidative stress, hippocampal neuronal damage and glial activation, and HMGB1/NF-κB signaling pathway proteins were also examined. Our results indicated that celastrol dose-dependently reduced hippocampal and serum concentration of pro-inflammatory markers (TNF-α, IL-1β, and IL-6) and oxidative stress marker (MDA), whereas the anti-inflammatory marker IL-10 and antioxidant markers (GSH, SOD, and CAT) were increased significantly in celastrol treated tGCI/R rats. Celastrol alleviated apoptotic neuronal death, inhibited reactive glial activation and proliferation and improved ischemia-induced neurological deficits. Simultaneously, we found that mechanisms responsible for the neuroprotective effect of celastrol could be attributed to its anti-inflammatory and antioxidant actions via inhibiting HMGB1/NF-κB signaling pathway. These findings provide a proof of concept for the further validation that celastrol may be a superior candidate for the treatment of severe cerebral ischemic patients in clinical practice in the future.

## Introduction

Stroke, also known as cerebrovascular accident is a major health problem worldwide (Villa et al., 2018), it is usually classified as ischemic, due to lack of blood flow, or hemorrhagic, due to bleeding (Koh and Park, 2017). Ischemic stroke accounts for around 85% of all stroke cases, and it is the leading cause of increased morbidity and mortality (Deng et al., 2017; Koh and Park, 2017). Transient global cerebral ischemia (GCI) may occur in a multitude of clinical settings including cardiac arrest (Frisch et al., 2017), severe hypotension and shock (Block, 1999; Wen et al., 2018), craniocerebral trauma (Veenith et al., 2016) and serious cerebrovascular accident (Johnston et al., 2019). Post-cardiac arrest brain injury affects more than 83% of people over 65 years old in United States (Jazayeri and Emert, 2019). Transient global ischemia would damage neurons in specific brain regions such as hippocampus in rodents and in human (Schmidt-Kastner, 2015) thus leading to neurological consequences including short- or long-term motor/cognitive dysfunction (Pulsinelli and Brierley, 1979; Li et al., 2018).

Tremendous preclinical trials confirmed that neuroprotective agents, thrombolysis and hypothermia may rescue brain damage (Koh and Park, 2017; Patel and McMullen, 2017). However, there are currently no effective pharmacological agents can promote the completely rehabilitation of patients with GCI (Li et al., 2018). Although thrombolytic therapy can reduce the neurological deficits caused by ischemic stroke, but there is a risk of bleeding after thrombolytic recanalization and further aggravating brain damage (Koh and Park, 2017). Therapeutic hypothermia (usually lowers head temperature to the range of 32–34°C) is the most potent therapeutic strategy that has been reported to benefit adult cardiac arrest patients, but it may cause severe side effects such as pneumonia, arrhythmia, seizures and metabolic and electrolyte disorders (Nielsen et al., 2011). Therefore, it is necessary to develop new therapeutic strategies to improve prognosis of ischemia induced brain injury. Neuroinflammation and oxidative stress play important roles in the pathogenesis of cerebral ischemia, numerous studies suggested that anti-inflammatory and/or anti-oxidant agents could alleviate brain damage and improve neurological outcome in stroke models (Peng et al., 2015; Chen T. et al., 2017). Hence, neuroinflammation inhibition and free radical ablation for prevention of post-ischemic brain injury is proposed to be useful in clinical settings.

High mobility group box 1 (HMGB1) is an important inflammatory mediator in ischemic brain (Shichita et al., 2014). HMGB-1 binds to its receptors such as toll like receptor 4 (TLR4) and receptors for advanced glycation end products (RAGE) thereafter activate nuclear factor-kappa B (NF-κB), which targets several pro-inflammatory genes including tumor necrosis alpha (TNF-α), interleukin 1beta (IL-1β) and interleukin 6 (IL-6), and eventually mediate neuroinflammation (Cai et al., 2016). Emerging studies showed that HMGB1 and NF-κB also participate in oxidative stress after cerebral ischemia (Tao et al., 2015).

Celastrol is a quinone methide triterpenoid compound extracted from traditional Chinese herb *Tripterygium wilfordii Hook. f.*, which is known to exert various biological and pharmacological effects including potent anti-inflammatory and anti-oxidant activities (Choi et al., 2016). Celastrol exerted beneficial effects in the treatment of neurodegenerative (Allison et al., 2001; Kiaei et al., 2005), autoimmune and inflammatory diseases (Venkatesha et al., 2016; Dai et al., 2019). One study demonstrated that celastrol protects rat kidney against acute ischemia-reperfusion injury (Chu et al., 2014). Celastrol could alleviate acute ischemic stroke-induced cerebral cortex inflammation damage by promoting microglia/macrophage M2 polarization (Jiang et al., 2018). However, whether celastrol possesses protective effect against tGCI/R through anti-inflammatory or antioxidant effect has not yet been fully elucidated. Thus, the current study was designed to examine the potential neuroprotective effects of celastrol on neuronal injury and behavioral deficits in tGCI/R rats, aiming to find new effective drug in the rescue of ischemic brain injury and to elucidate the hypothesis that HMGB1/NF-κB signaling pathway inhibition might be involved in the anti-inflammatory and anti-oxidative effects of celastrol.

## Materials and Methods

### Animals and Reagents

Healthy adult male Sprague-Dawley rats obtained from the Experimental Animal Research Center of Tongji Medical College, Wuhan, PR China (Certificate No. 42009800002519/SCXK(E)2016-0009) were used in the present study. Animals were maintained at specific pathogen-free and controlled conditions (relative humidity 50 ± 10% with ambient temperature 23 ± 1°C, 12/12 h light/darkness cycle) and supplied with tap water and pellet chow *ad libitum*. Rats were randomly assigned to experimental groups before receiving four vessel occlusion (4-VO) or sham surgery. Celastrol (MW 360.4) with purity greater than 98% was purchased from GuangRun Biotech co., LTD (Nanjing, China), a 200 mg/ml stock solution (−20°C for 3 months) was prepared in pure dimethyl sulfoxide (DMSO) and freshly diluted to indicated concentrations with peanut oil (47119, Sigma, St. Louis, MO, United States) before use.

All procedures were carried out strictly in accordance with the Guide for the Care and Use of laboratory Animals published by the National Institutes of Health (Publication No. 80-23, revised in 1996) and protocols were approved by the Animal Use and Care Committee for Laboratory Animals of Tongji Medical College.

### Transient Global Cerebral Ischemia Reperfusion (tGCI/R) Model and Celastrol Treatment

**FIGURE 1:**
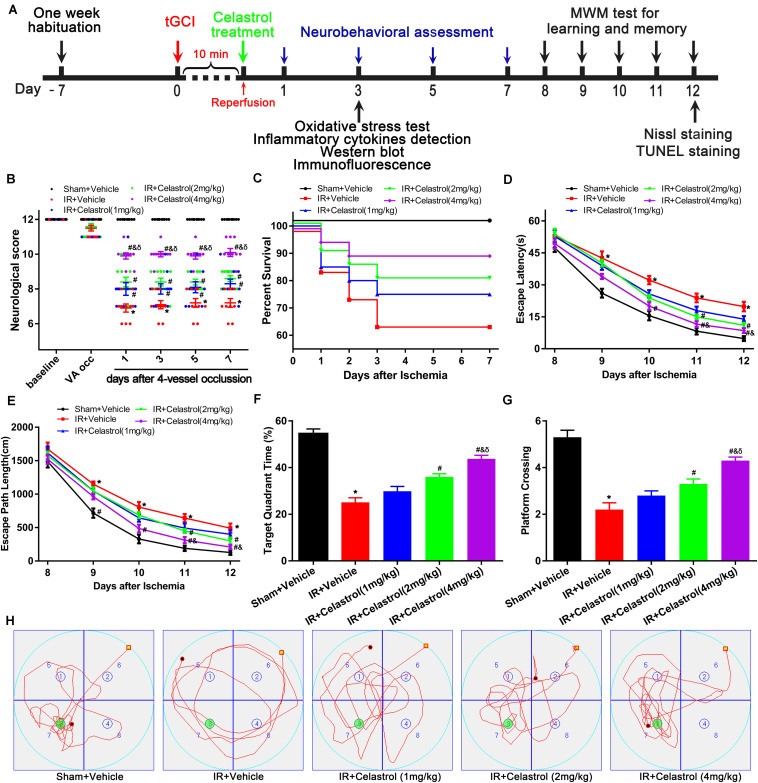
Experimental design and effect of celastrol treatment on ischemia induced neurobehavioral deficits. **(A)** Schematic diagram illustrating the experimental design of drug treatment and neurobehavioral assessment of ischemic cerebral injury. Seven days after habituation, rats were subjected to transient global cerebral ischemia reperfusion (tGCI/R), celastrol or vehicle was intraperitoneally injected immediately after reperfusion. Neurological score was evaluated at 1, 3, 7 days after tGCI/R and the (Morris Water Maze test) MWM was performed to assess learning and memory 7 days after tGCI/R, samples were collected 3 or 12 days after tGCI/R for further detection. **(B)** Neurological score shown for individual rats at different time points (*n* = 10 for each group) (Mann-Whitney-*U*-test). **(C)** Kaplan-Meier curves of animal survival for 7 days following vehicle or celastrol treatment after tGCI/R procedures. Values are expressed as percentages (*n* = 15, 20, 20, 20, 20 in Sham + Vehicle, IR + Vehicle, IR + Celastrol (1 mg/kg), IR + Celastrol (2 mg/kg) and IR + Celastrol (4 mg/kg) groups, respectively). **(D)** Mean escape latency of rats to reach the hidden platform during the place navigation test. **(E)** Mean escape path length of each rats to reach the hidden platform during the place navigation test. **(F)** Time spend in the target quadrant during the spatial probe test. **(G)** The number of times that rats crossed the original platform location during the spatial probe test. **(H)** Representative swimming trajectories of rats during the spatial probe test, green circles indicated the location of escape platform. The error bars represent mean ± S.E.M (*n* = 10, ^∗^*P* < 0.05 vs. Sham + Vehicle group; ^#^*P* < 0.05 vs. IR+ Vehicle group; ^&^*P* < 0.05 vs. IR + Celastrol (1 mg/kg) group; ^δ^ *P* < 0.05 vs. IR + Celastrol (2 mg/kg) group).

The experimental design was showed in Figure 1A. At the beginning of the experiment, a sample-size estimation was performed based on our historical data using SigmaStat (Systat Software, Inc., San Jose, CA, United States) with a power of 0.80. Based on this analysis, 130 rats were randomly divided into five groups after a 7-day habituation: Sham + Vehicle (*n* = 18), IR + Vehicle (*n* = 28), IR + Celastrol (1 mg/kg) (*n* = 28), IR + Celastrol (2 mg/kg) (*n* = 28) and IR + Celastrol (4 mg/kg) (*n* = 28). Animals in Sham and IR (model) groups were treated with vehicle (peanut oil containing 1% DMSO); IR + Celastrol group rats were treated with 1, 2, or 4 mg/kg celastrol intraperitoneally. All surgical procedures were conducted between 10:00 a.m. and 16:00 p.m. to minimize possible complications due to circadian rhythms. Transient GCI reperfusion model was induced by four-vessel occlusion (4-VO) method as reported by Pulsinelli and Brierley (Pulsinelli and Brierley, 1979) and previously used in our laboratory (Wei et al., 2017; Zhang et al., 2018, 2019). Briefly, fasted (12 h) rats were anesthetized with an intraperitoneal injection of 1.5% nembutal (45 mg/kg) and atropine sulfate (0.1 ml, 0.5 mg/ml), immobilized on an experimental bench with head till down 15° and bilateral vertebral arteries were permanent electro-coagulated using a monopolar microelectrode. Then both common carotid arteries (CCAs) were isolated and loosely assembled with surgical silk thread. Twenty-four hours later, rats were restraint after lightly anesthetized with isoflurane and both CCAs were re-exposed, the CCAs were occluded using noninvasive arterial clamps (FST #00400-03) for 10 min immediately after rats recovered from isoflurane anesthesia. Rats that lost righting reflex and showed mydriasis within 1 min after occlusion were selected for further study, animals that exhibited hoarseness or convulsion were excluded.

Celastrol or vehicle (mixture of DMSO and peanut oil) was administered i.p. immediately after the CCA aneurysm clamps were removed, the total volume of vehicle or drug solution is 1 ml. The dosages used for celastrol administration (1, 2, and 4 mg/kg) were selected based on preliminary studies (Li et al., 2012; Chu et al., 2014). Animals in Sham group were subjected to the same surgical process without VAs and CCAs occlusion. Body temperature was maintained at 37 ± 0.5°C throughout the whole surgical procedures using a feedback-regulated Homoeothermic Blanket Control Unit (Harvard Instruments, Natick, MA, United States) with a rectal temperature probe. The neck incision was disinfected, dressed with penicillin and 0.5% bupivacaine and then sutured. All rats were allowed to recover in a warming cage (37 ± 1°C) for about 4 h with free access to food and water.

### Measurement of Physiological Parameters

Physiological parameters were measured in all group rats before, during and after tGCI/R. Briefly, after anesthetized with nembutal on the first day, the left femoral artery was equipped with a heparinized polyethylene catheter for blood sample collection (150 μL), blood samples were collected 30 min of stabilization (baseline), 8 min after of ischemia (ischemia), and 30 min after reperfusion (recovery). Blood samples were then analyzed for pH, arterial oxygen saturation (SaO_2_), partial pressure of oxygen (PaO_2_) and carbon dioxide (PaCO_2_), blood glucose and hemoglobin (Hb) concentration using GEM 3000 Blood Gas Analyzer (GEM Premier 3000, United States).

### Neurological Deficits Evaluation and Survival Recording

Neurological outcome was assessed by an investigator who was blind to experimental design and animal groups using a standard neurological scoring system (Abella et al., 2004), with a maximum score of 12 (normal rat without neurological impairment) and minimum score of 0 (live rat but in a comatose status). The scoring system consists six indicators: level of consciousness, righting reflex, corneal reflex, respiration, coordination and movement/activity, and each indicator can be evaluated as 0, 1, or 2 point according to actual situation. The daily neurological score for each animal was calculated by summing the individual scores after completing evaluation. For survival rate study, all animals were monitored twice a day and survival status was recorded.

### Morris Water Maze Test

Morris water maze (MWM) tests were conducted for a consecutive 5 days to assess hippocampus dependent spatial learning and memory 7 days after tGCI/R as previously used (Jeffery and Morris, 1993; Vorhees and Williams, 2006; Zhang et al., 2018). The water maze (Institute of Materia Medica, Chinese Academy of Medical Sciences, China) consisted of an open black painted circular swimming tank (150 cm in diameter, 60 cm in height), placed in a darkened room and illuminated by dim light. The tank was divided into four equal quadrants labeled east (E), west (W), south (S), and north (N), partially filled with warm water (22 ± 2°C) and an escape platform (10 cm in diameter) was placed in a fixed location in target quadrant (SW) of the tank (marked with green circles in Figure 1H). Non-toxic black ink was added to the water so rats could not see the submerged platform. Visual cues (geometric figures: square, triangle, circle or star) that were visible from the tank were pasted on room walls. Animals were subjected to training trials (place navigation test, PNT) for five consecutive days, and followed by a probe test (spatial probe test, SPT) without escape platform. The experimenter blinded to the experimental design remained quiet and kept in a fixed location outside the tank during the test.

Before initial trial, rats were trained to stay on the escape platform for 30 s. During PNT, rats (*n* = 10) were trained to search the submerged escape platform (maximum 60 s) four times per day with 15 min intervals between each trial. Rats that failed to locate the platform within 60 s were manually guided to the platform and allowed to learn its location by staying 15 s on the platform. The average time or distance to find the hidden platform (escape latency or escape path length) were recorded using a video tracking system (Chinese Academy of Sciences, China), the learning ability of rats was evaluated by calculating the daily average escape latency or average escape path length from ten rats. Two hours after the final training trial, SPT was carried out to test short-term memory without escape platform and the time spend in target quadrant and the number of crossings over the original platform location were recorded.

### Enzyme-Linked Immunosorbent Assay (ELISA) for Inflammatory Cytokines Determination

Three days (72 h) after tGCI/R, hippocampal and serum levels of TNF-α, IL-1β, IL-6, and IL-10 were detected using rat specific ELISA kits (R&D Systems, Inc., MN, United States) in strict accordance with the manufacturer’s protocol (*n* = 4 per group). Briefly, after deep anesthetized with nembutal, blood samples were collected using heparin prefilled syringes from jugular vein and stored in heparin anticoagulant tube. The samples were centrifuged at 1360 g (3500 rpm) for 3 min at room temperature (23 ± 2°C) and then serum was taken for quantitative analysis of inflammatory cytokines (TNF-α, IL-1β, IL-6, and IL-10). After blood samples collection, animals were decapitated and brains were freshly removed. Bilateral hippocampi were quickly dissected on ice plate, one side was used for ELISA and Western blot, and the other was used for biochemical detection (MDA, GSH, SOD, and CAT). Rat hippocampi were homogenized using chilled RIPA buffer containing protease and phosphatase inhibitors, after 15 min centrifugation (12,000 g/10,444 rpm, 4°C) supernatants were collected using Eppendorf micropipette for further detection. Hippocampal or serum samples (10 μL) were added to specific 96-well ELISA plates, then 40 μL dilution buffer added and well mixed. Samples were incubated at room temperature for 60 min and washed three times with washing buffer, then the plates were dried with filter paper and added with zymolytes, after which the absorbance was measured at 450 nm using a spectrophotometer (SmartSpec Plus, Bio-Rad, United States). The readings were normalized and calculated according to the amount of protein or serum of each sample.

### Estimation of Malondialdehyde (MDA), Reduced Glutathione (GSH), Superoxide Dismutase (SOD), and Catalase (CAT) Level

As mentioned above, rat hippocampi were collected 3 days (72 h) after tGCI/R (*n* = 4) for determination of levels of MDA, GSH and activity of SOD and CAT using commercially available assay kits (Nanjing Jiancheng Bioengineering Co. Ltd, Nanjing, China) according to the manufacturer’s instruction. Briefly, hippocampal samples were quickly isolated and homogenized in ice-cold normal saline. After centrifugation with 1776 g (4000 rpm) for 10 min at 4°C, supernatants were collected using Eppendorf micropipette for further testing. The MDA (an indicator of lipid peroxidation) level was measured using thiobarbituric acid method and the absorbance at 532 nm was measured. The GSH content (an indicator of GPx activity) was measured according to microplate method and absorbance at 420 nm was measured. The SOD activity was evaluated using the hydroxylamine method and absorbance at 550 nm was measured. The CAT activity was assessed using ultraviolet method, with absorbance measured at 240 nm. The protein concentration of hippocampal samples were determined using the method of Bradford (Bradford, 1976).

### Western Blot Analysis

Total hippocampal protein of tGCI/R rats (*n* = 4) was extracted using chilled RIPA buffer containing protease inhibitors (PMSF) and phosphatase inhibitor cocktail with Teflon-glass homogenizer and centrifuged (12,000 g/10,444 rpm, 4°C) for 15 min. Nuclear and cytoplasmic protein (*n* = 4) was extracted using Nuclear and Cytoplasmic Protein Extract Kit (P0027, Beyotime, China) strictly referred to the manufacturer’s instructions. Protein concentrations were measured and adjusted using Bradford Protein Assay Kit (P0006, Beyotime, China) with bovine serum albumin as standard. Samples with an equal amount (30 μg/lane) were separated by precast 8–12% SDS-PAGE gels and then transferred onto 0.45 μm PVDF membranes (Roche, Switzerland) using Bio-Rad TransBlot apparatus. Membranes were blocked in 5% (w/v) fat-free dry milk at room temperature (23 ± 2°C) for about 1 h with gentle shaking, and then membranes were incubated overnight at 4°C with the following primary antibodies: rabbit anti-HMGB1 (1:1000; ab18256, Abcam), rabbit anti-TLR4 (1:1000; 19811-1-AP, Proteintech), rabbit anti-RAGE (1:1000; 16346-1-AP, Proteintech), rabbit anti-NF-κB p65 (1:1000, ab16502, Abcam), rabbit anti-p-NF-κB p65 (1:1000, 3033, CST), rabbit anti-IκB-α(1:1000, ab32518, Abcam), rabbit anti-p-IκB-α (1:1000, 9246, CST), rabbit anti-Bcl-2 (1:1000; 2876, CST), rabbit anti-Bax (1:1000; 2772, CST), rabbit anti-cleaved-Caspase-3 (1:1000; 9661, CST) or rabbit anti-Lamin B1 (1:500; BA1228, Boster), mouse anti-GAPDH (1:1000, BM3876, Boster) and mouse anti-β-actin (1:500, BM5422, Boster). After washing three times with 0.1% TBST, membranes were incubated with HRP-conjugated secondary antibody (1:5000, Boster) for 1 h at room temperature (23 ± 2°C), and then fully rinsed with 0.1% TBST (3 × 10 min). Finally, signals were visualized by Enhanced Chemiluminescence (ECL) kit and detected using ChemiDicTM XRS^+^ Imaging System (Bio-Rad, United States), band densities were analyzed using Image Lab 3.0 software (Bio-Rad, United States). The relative protein expression levels were corrected by using an internal reference control.

### Immunofluorescent Staining

Three days (72 h) after tGCI/R, rat brains (*n* = 4) were freshly removed and divided into two halves with a sharp blade on ice, one half was used for immunofluorescent analysis and the other was used for nuclear/cytoplasmic protein extraction. Immunofluorescent staining was performed as previously described in our lab (Zhang et al., 2018). Briefly, the whole half rat brain was wrapped with optimal cutting temperature compound (4583, SAKURA Tissue-Tek) and placed into −80°C pre-cooled isopentane for 2 min, then the frozen brains were cryosectioned (12 μm) using a microtome (CM1900, Leica, Germany) and mounted onto superfrost glass slides (Boster, Wuhan, China). Brain sections (AP:-2.8 −4.3 mm from Bregma (Paxinos and Watson, 1998)) were fixed using freshly prepared 4% paraformaldehyde for 10 min and blocked with 0.3% Triton X-100/10% normal goat serum at room temperature (23 ± 2°C). Then, the sections were incubated overnight at 4°C with the following primary antibodies: mouse anti-NeuN (1:100; MAB377, Merck Millipore), mouse anti-GFAP (1:800; MAB360, Merck Millipore), rabbit anti-Iba-1 (1:500; 019-19741, Wako), rabbit anti-HMGB1 (1:1000; ab18256, Abcam) or rabbit anti-NF-κB (1:500; ab16502, Abcam). Then sections were incubated with appropriate secondary antibodies (goat anti- mouse or rabbit) (Alexa Fluor, Molecular Probes Inc., United States) for 60 min at room temperature, and counterstained the nuclei with DAPI (1 ng/Nl; Boster) for 10 min. Slides were preserved with coverslips in mounting medium (50% glycerol aldehyde) after rinsed with 0.1 M PBS. Fluorescent microscopy (DM2500; Leica Microsystems) was used to examine the immunostained sections, six randomly selected microscopic fields from the hippocampal CA1 area were selected for further analyze.

### Nissl and Terminal Deoxynucleotidyl Transferase-Mediated dUTP-Biotin Nick End Labeling (TUNEL) Staining

After behavior test, Nissl and TUNEL staining were performed as previously described (Zhang et al., 2018). Briefly, rats (*n* = 4) were trans cardiac perfused with 500 ml 4°C pre-cooled 4% paraformaldehyde after perfusion with 300 ml heparinized cold saline (4°C) under deep anesthesia. Brains were carefully removed and post-fixed with 4% paraformaldehyde for 24 h at 4°C, dehydrated with gradient sucrose solution (10, 20, and 30%) until sank to the bottom. Then the brains were dried with filter paper and wrapped with optimal cutting temperature compound (4583, SAKURA Tissue-Tek) and cryosectioned using a microtome (CM1900, Leica, Germany). For histological detection, slices that containing the complete hippocampus (AP:-2.8 −4.3 mm from Bregma (Paxinos and Watson, 1998)) were selected. Brain sections were stained with 0.3% cresyl violet acetate (Sigma) for 30 min at room temperature, then the slides were washed with distilled water, dehydrated with gradient alcohol (70, 95, and 100%) for 5 min, and transparented with xylene. Sections were cover slipped with neutral balsam medium (36313ES60; Yeasen Biotechnology, China) and examined using light microscope (DM2500; Leica Microsystems, Germany) as described by Yrjanheikki et al. (1998), surviving neurons were measured using Image-Pro Plus version 6.0 (Media Cybernetics, United States) software by a blinded experimenter with reference to the method of Tian (Tian et al., 2014) and our previous study (Zhang et al., 2018) in the CA1 pyramidal cell layer, only whole neurons with visible nuclei were counted.

Next, brain slices were incubated with In Situ Cell Death Detection Kit (Roche Diagnostics, Indianapolis, United States) according to standard protocols. Briefly, sections were first incubated with TUNEL reaction buffer in a humidified chamber at 37°C for 1 h after treating with protease K for 20 min at room temperature (23 ± 2°C). Next, slides were rinsed and treated with converter-POD for 30 min at room temperature, apoptotic neurons were stained with diaminobenzidine substrate solution and total nuclei were counterstained with hematoxylin. Finally, sections were washed, dehydrated in graded ethanol (70, 95, and 100%) and transparented with xylene, coverslipped with neutral balsam medium (36313ES60; Yeasen Biotechnology, China) and observed with a light microscope (DM2500; Leica Microsystems, Germany). For quantitative analyses, every sixth section of the hippocampus was processed and pixels of 0.10 mm^2^ were visualized by an examiner blinded to experimental design and group assignment, the total number of TUNEL positive cells (dyed brown) was counted using Image-Pro Plus version 6.0 (Media Cybernetics, United States) software and expressed as cells/mm^2^. The apoptotic index was calculated and expressed as a percentage of the total number of neurons in which TUNEL positive neurons were present.

### Statistical Analysis

All data were analyzed using GraphPad Prism 7.0 (Graphpad Software Inc., San Diego, CA, United States) and values are shown as mean ± SEM. Rat survival rate was analyzed using Kaplan-Meier method with data compared by log-rank test. Neurological deficit scores were analyzed using Mann-Whitney *U*-test followed by Bonferroni correction. Data obtained from the Morris Water Maze task were analyzed by repeated-measures of two-way analysis of variance (ANOVA) (treatment × day) or one-way ANOVA followed by Bonferroni test for multiple *post-hoc* comparisons. Other data were subjected to one-way ANOVA followed by Bonferroni *post-hoc* comparison as needed. All hypotheses were tested at a significance level of 0.05.

## Results

### Physiological Parameters

Values for pH, PaO_2_, PaCO_2_, SaO_2_, blood glucose and Hb concentration were within the normal range and differences between groups were not statistically significant (*P* > 0.05) (Table 1).

**TABLE 1:** Physiological parameters during ischemia/reperfusion period in rats.

Parameter	Time	Sham+Vehicle	IR+Vehicle	IR+Celastrol (1 mg/kg)	IR+Celastrol (2 mg/kg)	IR+Celastrol (4 mg/kg)
pH	Baseline	7.42 ± 0.01	7.41 ± 0.02	7.41 ± 0.01	7.41 ± 0.01	7.42 ± 0.01
	Ischemia	7.39 ± 0.02	7.39 ± 0.01	7.38 ± 0.01	7.38 ± 0.01	7.39 ± 0.01
	Reperfusion	7.40 ± 0.01	7.37 ± 0.02	7.37 ± 0.02	7.39 ± 0.02	7.39 ± 0.02
PaO_2_ (mm Hg)	Baseline	86.0 ± 6.4	84.8 ± 6.3	85.3 ± 5.9	85.2 ± 5.7	85.3 ± 5.2
	Ischemia	85.8 ± 6.3	85.5 ± 5.4	84.5 ± 6.1	84.6 ± 6.1	85.3 ± 6.1
	Reperfusion	85.7 ± 5.8	85.4 ± 5.9	85.1 ± 5.8	85.3 ± 5.5	84.7 ± 5.6
PaCO_2_ (mm Hg)	Baseline	34.7 ± 0.7	34.4 ± 0.6	35.4 ± 0.8	35.7 ± 0.7	36.2 ± 0.8
	Ischemia	35.1 ± 0.8	36.3 ± 0.9	34.7 ± 0.7	35.1 ± 0.9	36.6 ± 0.9
	Reperfusion	37.4 ± 0.8	36.0 ± 0.7	35.7 ± 0.6	36.3 ± 0.6	35.9 ± 0.7
SaO_2_ (%)	Baseline	98.1 ± 4.4	97.6 ± 4.2	98.1 ± 4.4	97.4 ± 4.7	98.1 ± 4.4
	Ischemia	97.5 ± 5.1	97.5 ± 5.3	97.7 ± 5.1	98.3 ± 5.2	97.7 ± 5.6
	Reperfusion	97.9 ± 3.9	97.4 ± 5.7	97.4 ± 3.8	97.8 ± 4.8	97.3 ± 4.9
Glucose (mmol/L)	Baseline	4.75 ± 0.75	4.68 ± 0.69	4.76 ± 0.71	4.92 ± 0.47	4.82 ± 0.67
	Ischemia	4.97 ± 0.71	4.77 ± 0.76	4.83 ± 0.59	4.83 ± 0.58	4.78 ± 0.58
	Reperfusion	4.67 ± 0.62	4.81 ± 0.72	4.92 ± 0.74	4.90 ± 0.64	4.69 ± 0.63
Hb (g/dL)	Baseline	11.4 ± 0.9	11.0 ± 0.8	11.0 ± 0.8	10.8 ± 0.8	11.1 ± 0.7
	Ischemia	10.4 ± 0.8	10.5 ± 0.9	10.6 ± 0.7	10.6 ± 0.8	11.0 ± 0.9
	Reperfusion	10.6 ± 0.7	10.8 ± 0.8	10.5 ± 0.7	10.7 ± 0.9	11.0 ± 0.8

**All physiological variables were measured at 30 min of stabilization (baseline), after 8 min of ischemia (ischemia), and after 30 min of reperfusion (recovery).**

### Celastrol Improves Neurological Outcome and Increases Survival Rate

Transient GCI leads to measurable changes in neurological deficit scores. As shown in Figure 1B, 4-VO induced obviously neurological deficit in vehicle treated tGCI/R rats, with neurological score decreased to 7 points when compared with sham-operated rats (12 points). However, neurological score was improved in celastrol treated tGCI/R rats with the most obvious improvement effect in 4 mg/kg group, the neurological scores were fairly stable in all groups during the 7-days after 4-VO. Vertebral-artery occlusion caused consistent mild ataxia in all tGCI/R rats (neurological score reduced to 11) as expected. However, celastrol dose-dependently improved neurological outcome of tGCI/R rats when compared to their littermates in vehicle treated tGCI/R groups in the same time points. Mann-Whitney-*U*-test revealed a significant difference between vehicle treated tGCI/R rats and cleastrol treated tGCI/R rats (all *P* < 0. 05).

We initially included a total of 99 rats for survival analysis and finally excluded 9 rats due to unsuccessful ischemia (4/99), hoarseness (2/99) and convulsion (3/99). As shown in Figure 1C, there were no rats died in Sham + Vehicle group during the seven-observation days, the 7-days survival rate increased in tGCI/R rats treated with celastrol (75, 80, and 90%for 1, 2, and 4 mg/kg) when compared with vehicle treated tGCI/R rats (65%), but the differences were not statistically significant (χ^2^ = 6.6, *P* = 0.16). However, these results still prompt us to speculate that celastrol treatment immediately after ischemia-reperfusion may protect rats from death resulting from tGCI/R.

### Celastrol Improves tGCI/R Induced Behavioral Deficits in Morris Water Maze

We examined animal performance in Morris Water Maze (MWM) 7 days after tGCI/R. During PNT, there were no significant differences among rats as for average swimming speed (data not shown), whereas escape latency and escape path length showed a decreased trend during the five-training days (Figures 1D,E). Repeated measures of two-way ANOVA for escape latency revealed significant main effect [treatment *F*_(4, 225)_ = 40, *P* < 0. 01; day *F*_(4, 225)_ = 389, *P* < 0.01; interaction *F*_(16, 225)_ = 1.2, *P* = 0.25]. As for escape path length, there was also significant main effect [treatment *F*_(4, 225)_ = 28, *P* < 0. 01; day *F*_(4, 225)_ = 348, *P* < 0.01; interaction *F*_(16, 225)_ = 0.76, *P* = 0.73]. Compared to sham rats, vehicle treated tGCI/R rats required longer time and exhibited longer path length searching for the platform during PNT (Figures 1D,E). Whereas celastrol treated tGCI/R rats performed better when compared to vehicle treated tGCI/R rats as reflected by significant reduction in escape latency and escape path length (all *P* < 0.05).

During SPT, time spend in target quadrant (Figures 1F,H) and platform crossings (Figures 1G,H) of celastrol treated tGCI/R rats were significantly higher than that of vehicle treated tGCI/R rats. The mean target quadrant time for each group was 55 ± 6.6, 25 ± 6.3, 30 ± 6.5, 36 ± 4.4, 44 ± 4.7 s and the mean platform crossing number for each group was 5.3 ± 0.95, 2.2 ± 0.92, 2.8 ± 0.63, 3.3 ± 0.67, 4.3 ± 0.48. One-way ANOVA of target quadrant time [*F*_(4, 45)_ = 41, *P* < 0.01] and platform crossings [*F*_(4, 45)_ = 27, *P* < 0.01] showed significant treatment effect. Furthermore, total distance traveled in target quadrant (Figure 1H) was shorter for vehicle treated tGCI/R rats, while longer for celastrol treated tGCI/R rats.

### Therapeutic Effect of Celastrol on tGCI/R Induced Apoptotic Neuronal Death

**FIGURE 2:**
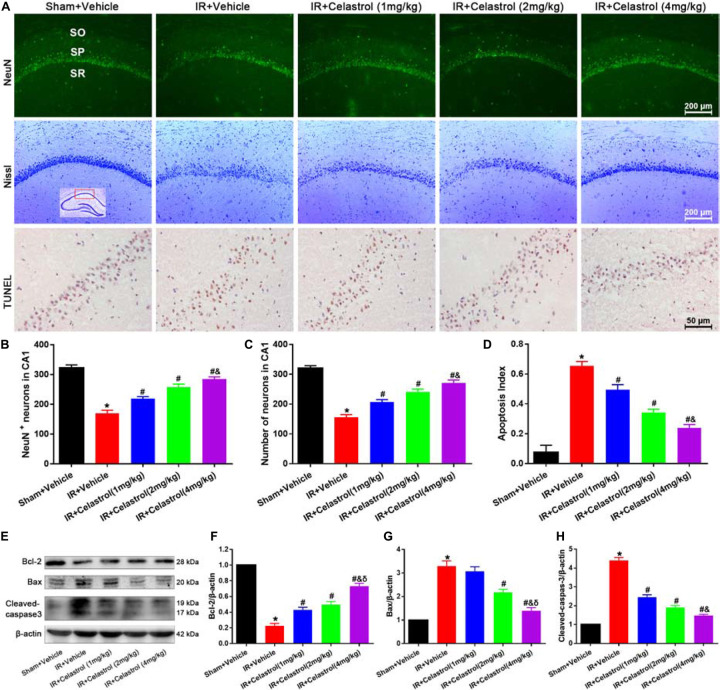
Celastrol inhibits apoptotic hippocampal neuronal death. **(A)** Representative images of NeuN immunofluorescent staining sections (first row), cresyl violet-stained sections (second row) and TUNEL-stained sections (third row) in hippocampal CA1 region of tGCI/R rats, the built-in schematic diagram with red rectangular shows the area that we analyzed. Data were obtained from 4 independent animals, and the results of a typical experiment are exhibited. Scale bar = 200 μm. SO, stratum orients; SP, stratum pyramidal; SR, stratum radium. **(B)** Quantitative analysis of NeuN positive neurons. **(C)** Quantitative analysis of Nissl-positive neurons **(D)** The apoptosis index of hippocampal CA1 neurons. **(E–H)** Western blot analysis of apoptosis-related proteins Bcl-2, Bax and cleaved-caspase-3, β-actin was used as an internal control. The error bars represent mean ± S.E.M (*n* = 4, ^∗^*P* < 0.05 vs. Sham + Vehicle group; ^#^*P* < 0.05 vs. IR+ Vehicle group; ^&^*P* < 0.05 vs. IR + Celastrol (1 mg/kg) group; ^δ^ *P* < 0.05 vs. IR + Celastrol (2 mg/kg) group).

We first detected hippocampal neuronal loss by NeuN immunofluorescencent-staining (Figure 2A, first row). Statistical results showed that number of NeuN-positive cells in CA1 of vehicle treated tGCI/R rats (170 ± 10) was significantly reduced when compared to sham rats (323 ± 10) (*P <* 0.05). However, celastrol rescued the reduction of NeuN-positive neurons induced by tGCI/R, with 219 ± 6.7, 258 ± 9.5 and 285 ± 7.0 in 1, 2, and 4 mg/kg treated tGCI/R rats (all *P* < 0.01 vs. IR + Vehicle) (Figure 2B). We next detected neuronal damage by Nissl-staining (Figure 2A, second row), pyramidal neurons with neatly arrange and relative regular shape were seen in sham rats, whereas pyramidal neurons were arranged randomly with smaller cell body in vehicle treated tGCI/R rats. Moreover, celastrol dose-dependently rescued tGCI/R induced pyramidal neuron damage. The number of Nissl^+^ neurons in vehicle treated tGCI/R rats (156 ± 8.6) was significantly less than that of sham rats (320 ± 8.9, *P* < 0.05), whereas celastrol significantly alleviated tGCI/R induced neuronal loss, with 207 ± 8.1, 241 ± 9.3, and 272 ± 9.3 in 1, 2, and 4 mg/kg treated tGCI/R rats (all *P* < 0.05 vs. IR + Vehicle) (Figure 2C).

We further performed TUNEL-staining to detect apoptotic neuronal death (Figure 2A, third row). Neurons with normal morphology were observed in CA1 of sham rats. However, TUNEL-positive neurons were increased in vehicle treated tGCI/R rats and celastrol markedly decreased TUNEL-positive neurons number in tGCI/R rats. Statistic results showed that the apoptosis index of vehicle treated tGCI/R rats (0.66 ± 0.03) was significantly higher than that of sham rats (0.08 ± 0.05), and this was partially diminished by celastrol, with 0.50 ± 0.03, 0.34 ± 0.02 and 0.24 ± 0.02 in 1, 2, and 4 mg/kg treated tGCI/R rats (all *P* < 0.01 vs. IR + Vehicle) (Figure 2D).

We also assessed whether apoptotic responses were related to the protective effects of celastrol. As shown in Figures 2E–H, in vehicle treated tGCI/R rats, hippocampal level of Bax was increased but Bcl-2 level was decreased when compared to sham rats, whereas these effects were reversed by celastrol (all *P* < 0.01 vs. IR + Vehicle). Moreover, the activation of caspase-3 was significantly ameliorated by celastrol.

### Therapeutic Effect of Celastrol on tGCI/R Induced Glial Activation

**FIGURE 3:**
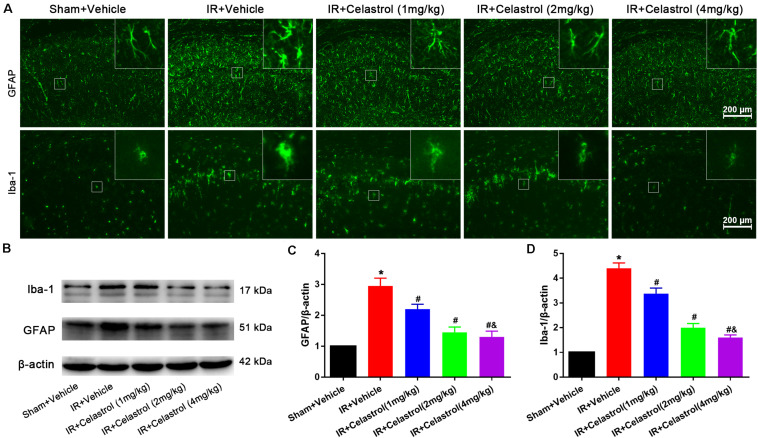
Celastrol attenuates glial activation in the hippocampus. **(A)** Examples of immunofluorescent images of GFAP and Iba-1 staining in rat hippocampus 3 days after tGCI/R, the built-in thumbnails in the upper right corner show the morphological changes of astrocytes and microglia. Data were obtained from 4 independent animals, and the results of a typical experiment are presented. Scale bar = 200 μm. **(B–D)** Representative western-blot images and quantitative analysis of GFAP and Iba-1 in the hippocampus 3 days after tGCI/R. The error bars represent mean ± SEM (*n* = 4, *P < 0.05 vs. Sham + Vehicle group; ^#^*P* < 0.05 vs. IR+ Vehicle group; ^&^*P* < 0.05 vs. IR + Celastrol (1 mg/kg) group; ^δ^ *P* < 0.05 vs. IR + Celastrol (2 mg/kg) group).

Immunofluorescence and immunoblotting were used to assess expression characters of GFAP and Iba-1. Astrocytes in vehicle treated tGCI/R rats showed hypertrophic cell body and shorter protrusions when compared to astrocytes with small body and slender protrusions in sham rats, whereas celastrol dose-dependently inhibit tGCI/R induced astrocyte activation (Figure 3A, first row). Immunoblotting (Figures 3B,C) showed that relative GFAP level in vehicle treated tGCI/R rats (3.0 ± 0.20) was significantly increased when compared to sham rats (set as 1) (*P* < 0.05). Whereas celastrol dose-dependently reversed tGCI/R induced GFAP level increase, with 2.2 ± 0.16, 1.5 ± 0.17, and 1.3 ± 0.19 for 1, 2, and 4 mg/kg treated tGCI/R rats (all *P* < 0.01 vs. IR + Vehicle). Iba-1 immunofluorescent staining (Figure 3A, second row) showed that tGCI/R induced microglia activation (hypertrophic cell body with fewer branches). However, celastrol treatment dose-dependently rescued tGCI/R induced morphological changes of microglia. Immunoblotting result indicated that increased Iba-1 level could partially be reversed by celastrol, with 3.4 ± 0.23, 2.0 ± 0.17, and 1.6 ± 0.11 for 1, 2, and 4 mg/kg treated tGCI/R rats and 4.4 ± 0.22 for vehicle treated tGCI/R rats (Figures 3B,D).

### Therapeutic Effect of Celastrol on Inflammatory Mediators After tGCI/R

**FIGURE 4:**
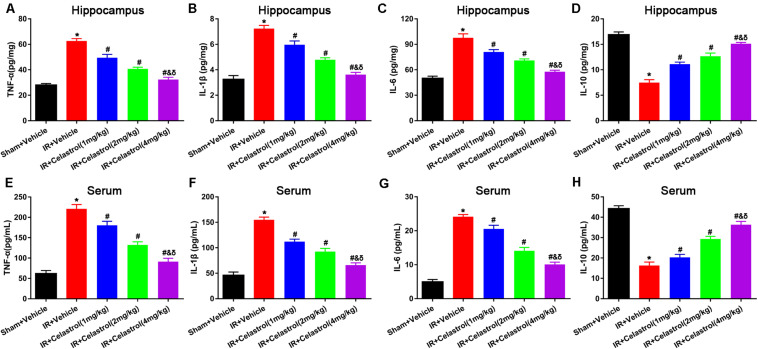
Effects of celastrol on hippocampal and serum levels of TNF-α, IL-1β,.IL-6 and IL-10. **(A)** Hippocampal TNF-α level in vehicle or celastrol treated sham or tGCIR rats. **(B)** Hippocampal IL-1β level in vehicle or celastrol treated sham or tGCIR rats. **(C)** Hippocampal IL-6 level in vehicle or celastrol treated sham or tGCIR rats. **(D)** Hippocampal IL-10 level in vehicle or celastrol treated sham or tGCIR rats. **(E)** Serum TNF-α level in vehicle or celastrol treated sham or tGCIR rats. **(F)** Serum IL-1β level in vehicle or celastrol treated sham or tGCIR rats. **(G)** Serum IL-6 level in vehicle or celastrol treated sham or tGCIR rats. **(H)** Serum IL-10 level in vehicle or celastrol treated sham or tGCIR rats. The error bars represent mean ± SEM (*n* = 4, *P < 0.05 vs. Sham + Vehicle group; ^#^ *P* < 0.05 vs. IR+ Vehicle group; ^&^ *P* < 0.05 vs. IR + Celastrol (1 mg/kg) group; ^δ^ *P* < 0.05 vs. IR + Celastrol (2 mg/kg) group).

Inflammatory cytokines were determined by detecting protein level of TNF-α, IL-1β, IL-6, and IL-10 in the hippocampus and serum using ELISA. As shown in Figures 4A–C, hippocampal levels of TNF-α, IL-1β, and IL-6 in vehicle treated tGCI/R rats were dramatically enhanced, which were 2. 25-, 2. 25-, and 1.96-fold higher than those of sham rats. On the contrary, hippocampal IL-10 level (Figure 4D) reduced to 44% of sham rats. Celastrol (1, 2, and 4 mg/kg) dose-dependently mitigated the accumulation of pro-inflammatory cytokines induced by tGCI/R, hippocampal levels of TNF-α were 79, 65, 51% of that in vehicle treated tGCI/R rats, hippocampal levels of IL-1β were 83, 67, 50% of that in vehicle treated tGCI/R rats, and hippocampal levels of IL-6 were 83, 72, 59% of that in vehicle treated tGCI/R rats. Simultaneously, celastrol reversed the reduction of hippocampal IL-10 level, which was 1. 47-, 1. 73-, and 2.0- fold higher than that of vehicle treated tGCI/R rats. Serum levels of inflammatory mediators were consistent with that of hippocampus (Figures 4E–H). The serum levels of TNF-α, IL-1β, and IL-6 in vehicle treated tGCI/R rats were 3. 6-, 3. 4-, 4.9-fold than that of sham rats, but serum IL-10 level reduced to 36% of sham rats. Celastrol (1, 2, and 4 mg/kg) partially reversed the changing trend of serum pro-inflammatory cytokines, levels of serum TNF-α were 82, 60, 41% of that in vehicle treated tGCI/R rats, levels of serum IL-1β were 72, 60, 43% of that in vehicle treated tGCI/R rats, and levels of serum IL-6 were 88, 58, 42% of that in vehicle treated tGCI/R rats, whereas serum IL-10 level was 1. 25-, 1. 81-, and 2.25-fold higher than that of vehicle treated tGCI/R rats.

### Therapeutic Effect of Celastrol on tGCI/R Induced Oxidative Stress

**FIGURE 5:**
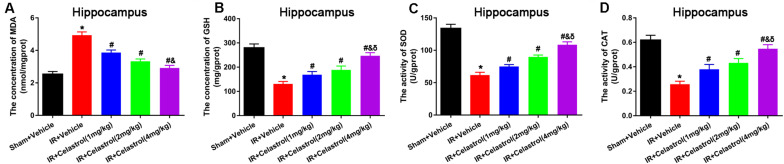
Celastrol alleviates tGCI/R induced oxidative stress in the hippocampus. **(A)** The hippocampal MDA level in vehicle or celastrol treated sham or tGCIR rats. **(B)** The hippocampal GSH level in vehicle or celastrol treated sham or tGCIR rats. **(C)** The hippocampal SOD activity in vehicle or celastrol treated sham or tGCIR rats. **(D)** The hippocampal CAT activity in vehicle or celastrol treated sham or tGCIR rats. The error bars represent mean ± SEM (*n* = 4, *P < 0.05 vs. Sham + Vehicle group; ^#^*P* < 0.05 vs. IR+ Vehicle group; ^&^*P* < 0.05 vs. IR + Celastrol (1 mg/kg) group; ^δ^ *P* < 0.05 vs. IR + Celastrol (2 mg/kg) group).

Malondialdehyde (MDA) and glutathione (GSH) level, superoxide dismutase (SOD), and catalase (CAT) activity were measured in hippocampal homogenates. As shown in Figures 5A–D, massive increase in MDA level (4.90 ± 0.20 vs. 2.50 ± 0.17) coinciding with massive decrease in GSH level (131.00 ± 10.00 vs. 280.00 ± 16.00), activities of SOD (62.00 ± 4.20 vs. 134 ± 6.40) and CAT (0.26 ± 0.03 vs. 0.62 ± 0.04) were decreased in vehicle treated tGCI/R rats when compared to sham rats, whereas celastrol (1, 2, 4 mg/kg) treatment resulted in a lower MDA content (3.90 ± 0.15, 3.30 ± 0.15, 2.90 ± 0.16) and higher GSH content (169.00 ± 13.00, 189.00 ± 16.00, 247.00 ± 13.00), and enhanced activities of SOD (75.00 ± 2.90, 90.00 ± 2.90, 109.00 ± 4.30) and CAT (0.38 ± 0.04, 0.43 ± 0.04, 0.55 ± 0.03) as compared to vehicle treated tGCI/R rats.

### Celastrol Decreased HMGB-1, TLR4 and RAGE Level and Rescued HMGB-1 Translocation

**FIGURE 6:**
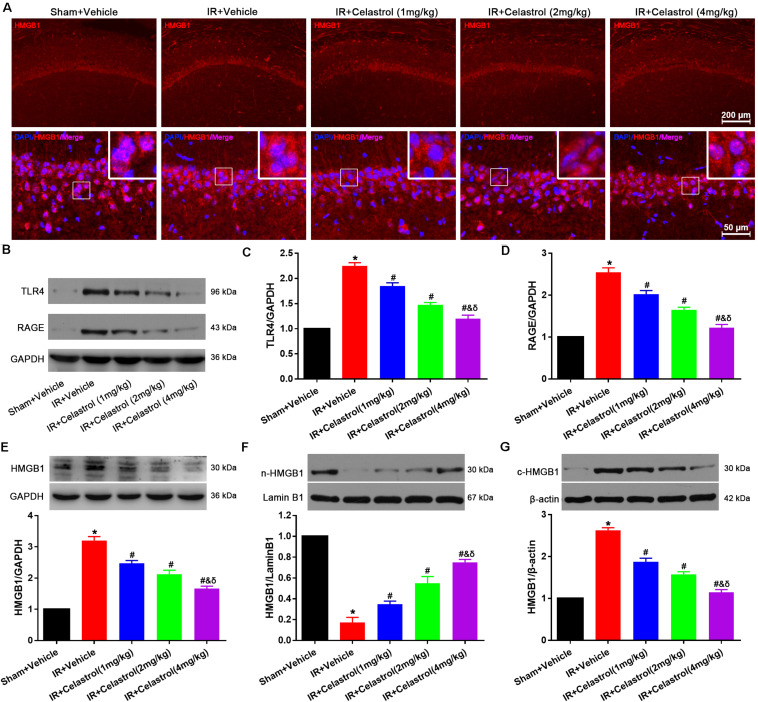
Celastrol decreased HMGB-1, TLR4 and RAGE expression and rescued HMGB-1 translocation. **(A)** Representative immunofluorescent images of HMGB-1 in the hippocampus 3 days after tGCI/R, celastrol partially reversed tGCI/R induced HMGB1 nuclear-cytoplasm translocation. The upper right corner built-in thumbnails show the nuclear-cytoplasmic translocation of HMGB-1. Data were obtained from 4 independent animals, and the results of a typical experiment are presented. Red: HMGB1, blue: DAPI and scale bar = 200 μm. **(B–D)** Representative western-blot images and quantitative analysis of TLR4 and RAGE in the hippocampus 3 days after tGCI/R. **(E–G)** Representative western-blot images and quantitative analysis of total HMGB1, nuclear HMGB1 and cytoplasmic total HMGB1 in the hippocampus 3 days after tGCI/R. The error bars represent mean ± SEM (*n* = 4, *P < 0.05 vs. Sham + Vehicle group; ^#^*P* < 0.05 vs. IR+ Vehicle group; ^&^*P* < 0.05 vs. IR + Celastrol (1 mg/kg) group; ^δ^ *P* < 0.05 vs. IR + Celastrol (2 mg/kg) group).

HMGB1 expression status was detected by immunofluorescence (Figure 6A), HMGB1 signal was enhanced and the nucleus translocation was observed in vehicle treated tGCI/R rats, whereas celastrol reversed this change. To confirm this, Western-blot was used to detect the level of total HMGB1, TLR4, and RAGE. As shown in Figures 6B–E, levels of total HMGB-1, TLR4 and RAGE in vehicle treated tGCI/R rats (3.2 ± 0.13, 2.3 ± 0.07, and 2.6 ± 0.10) were notably increased when compared with sham control (set as 1) (all *P* < 0.01), whereas celastrol dose-dependently reversed the upregulation of these three protein, with 2.5 ± 0.09, 2.1 ± 0.13, 1.7 ± 0.08 for total HMGB1, 1.9 ± 0.07, 1.5 ± 0.05 and 1.2 ± 0.07 for TLR4 and 2.0 ± 0.09, 1.7 ± 0.07, and 1.2 ± 0.08 for RAGE in 1, 2, and 4 mg/kg treated tGCI/R rats (all *P* < 0.01 vs. IR + Vehicle). In addition, nuclear HMGB-1 level (0.18 ± 0.05) was markedly decreased accompanied with a markedly increase in cytosolic HMGB-1 level (2.6 ± 0.06) in vehicle treated tGCI/R rats when compared with sham rats (all *P* < 0.01), which was significantly rehabilitated by celastrol, with 0.35 ± 0.03, 0.55 ± 0.07, 0.75 ± 0.03 for nuclear HMGB-1, 1.9 ± 0.09, 1.6 ± 0.06, and 1.2 ± 0.07 for cytosolic HMGB1 in 1, 2, and 4 mg/kg treated tGCI/R rats (all *P* < 0.01 vs. IR + Vehicle) (Figures 6F,G).

### Celastrol Inhibited NF-κB Upregulation and Nuclei Translocation

**FIGURE 7:**
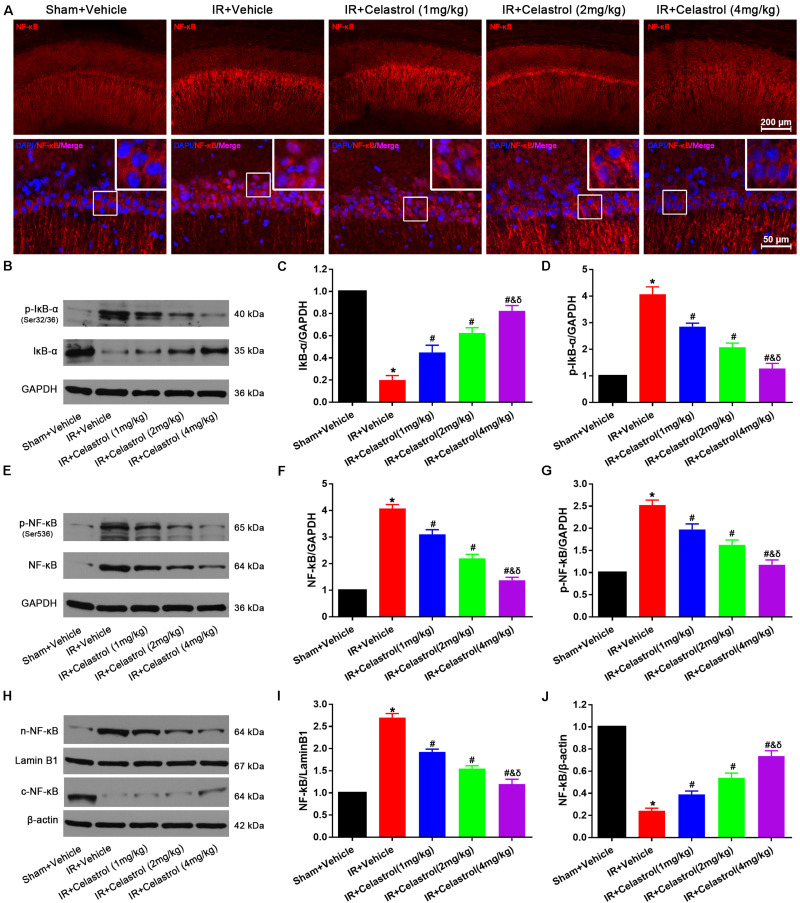
Celastrol inhibited NF-κB upregulation and nuclei translocation. **(A)** Representative immunofluorescent images of NF-κB in the hippocampus 3 days after tGCI/R, celastrol partially rescued tGCI/R induced NF-κB cytoplasmic-nuclear translocation. The upper right corner built-in thumbnails show the cytoplasmic-nuclear translocation of NF-κB. Data were obtained from four independent animals, and the results of a typical experiment are presented. Red: NF-κB, blue: DAPI scale bar = 200 μm. **(B–D)** Representative western-blot images and quantitative analysis of IκBα and phosphorylated IκBα in the hippocampus 3 days after tGCI/R. **(E–G)** Representative western-blot images and quantitative analysis of total NF-κB and phosphorylated NF-κB in the hippocampus 3 days after tGCI/R. **(H–J)** Representative western-blot images and quantitative analysis of nuclear and cytoplasmic NF-κB in the hippocampus 3 days after tGCI/R. The error bars represent mean ± SEM (*n* = 4, *P < 0.05 vs. Sham + Vehicle group; ^#^*P* < 0.05 vs. IR+ Vehicle group; ^&^*P* < 0.05 vs. IR + Celastrol (1 mg/kg) group; ^δ^ *P* < 0.05 vs. IR + Celastrol (2 mg/kg) group).

NF-κB expression status was examined by immunofluorescent staining (Figure 7A), few cells were stained with NF-κB p65 in hippocampal CA1, and positive signals are mainly concentrated in cytoplasm in sham rats. However, NF-κB p65 positive signals in vehicle treated tGCI/R rats were mainly located at nucleus. Celastrol dose-dependently reduced positive nuclei NF-κB p65 signal whereas enhanced cytoplasmic NF-κB p65 signal. We further quantified the level of IκB-α, phosphorylated IκB-α, total NF-κB p65, phosphorylated NF-κB p65 and cytosolic/nuclear NF-κB p65 using Western-blot. Relative level of IκB-α in vehicle treated tGCI/R rats (0.20 ± 0.04) was significantly lower than that of sham rats (set as 1), whereas celastrol dose-dependently reversed the downregulation of IκB-α, with 0.45 ± 0.07, 0.63 ± 0.07 and 0.83 ± 0.05 in 1, 2, and 4 mg/kg treated tGCI/R rats (all *P* < 0.05 vs. IR + Vehicle) (Figures 7B,C). The change of phosphorylated IκB-α was just the opposite of IκB-α, with 4.08 ± 0.27 in vehicle treated tGCI/R rats and 2.85 ± 0.13, 2.08 ± 0.15 and 1.28 ± 0.19 in 1, 2, and 4 mg/kg treated tGCI/R rats (Figures 7B,D). Total level of NF-κB p65 and phosphorylated NF-κB p65 were increased in vehicle treated tGCI/R rats when compared to sham rats, but celastrol inhibited the increase of NF-κB p65 and phosphorylated NF-κB p65 induced by tCCI/R (Figures 7E–G). In addition, NF-κB p65 level in vehicle treated tGCI/R rats was significantly increased in nuclear fractions but concurrently decreased in cytosol, indicating the translocation of NF-κB from cytosol to nucleus, However, celastrol dose-dependently reversed the nuclear/cytosolic translocation of NF-κB p65 (Figures 7H–J).

## Discussion

**FIGURE 8:**
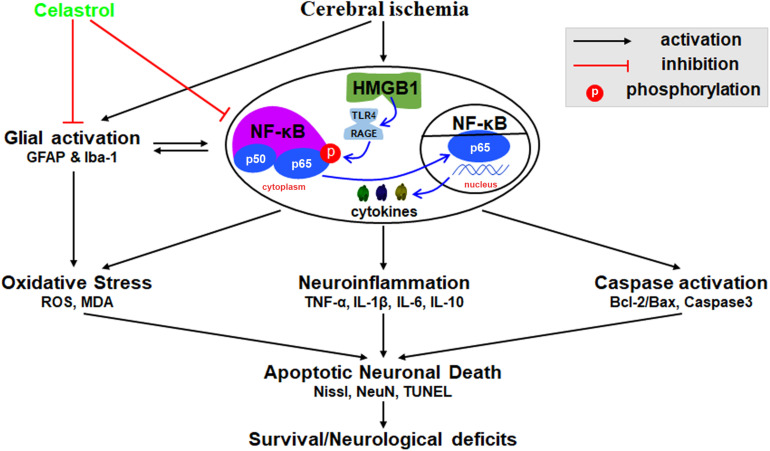
Schematic diagram illustrating possible neuroprotective mechanisms of celastrol against glial activation, oxidative stress and neuroinflammation in tGCI/R rats via the high mobility group box 1 (HMGB1) and Toll-like receptor 4 (TLR4) /nuclear factor (NF-κB) pathway. Cerebral ischemia induced HMGB1 nuclear-cytoplasmic translocation and activate TLR4/RAGE, promote the phosphorylation of IκBα, thus inducing the translocation of NF-κB from the cytoplasm to the nucleus, promote gene expression and generate a series of cytokines, thus promoting glial activation, neuroinflammation, oxidative stress, and caspase activation, eventually lead to apoptotic neuronal death. GFAP, glial fibrillary acid protein; Iba-1, ionized calcium binding adaptor molecular 1; ROS, reactive oxygen species; MDA, malondialdehyde; TNF-α, tumor necrosis factor α; IL-β, interleukin-β; IL-6, interleukin-6; IL-10, interleukin-10.

We herein demonstrated that plant-derived celastrol treatment after tGCI/R could partially inhibit glial activation and proliferation, attenuate neuroinflammation, mitigate oxidative stress, rescue hippocampal apoptotic neuronal death and improve neurological outcome and survivals. The mechanism may involve the inhibition of HMGB1/NF-κB signaling pathway (Figure 8).

Celastrol is a traditional Chinese herbal with potential anti-inflammatory and anti-oxidative effects (Bian et al., 2016), it could alleviate inflammation by inhibiting the release of TNF-α and IL-1β from microglia after amyloid-β_1__–__42_ stimulation *in vitro* (Veerappan et al., 2017). Previous studies showed that celastrol may exert beneficial effects on myocytes through anti-inflammation and anti-oxidative actions in hypertensive rats (Yu et al., 2010) and alleviate neurological deficits of middle cerebral artery occlusion (MCAO) rats (Li et al., 2012). Here we indicated that celastrol exerted neuroprotective effects through anti-inflammatory and anti-oxidant reaction in tGCI/R rats. Recent studies suggested celastrol has narrow therapeutic window (Konieczny et al., 2014) and showed high mortality (40%) in mice with 4 mg/kg administration (Luo et al., 2017) (Raja et al., 2011), whereas we found that celastrol (4 mg/kg) still showed better neuroprotective effects, i.e., 4 mg/kg celastrol can increase survival rate and improve cognitive ability of tGCI/R rats, we speculate this discrepancy may be attributed to animal strain and administration time.

Vertebral-artery occlusion caused consistent mild ataxia in all tGCI/R rats (neurological score reduced to near 11 points) as a previous study (Akdemir et al., 2014), and we deduce this may be attributed to reduced cerebellar blood flow owing to vertebral artery occlusion. Hippocampus is principally involved in spatial learning and declarative memory (Morris et al., 1982; Sadelli et al., 2017). Hippocampal neurons are sensitive to ischemia (Pulsinelli and Brierley, 1979; Kim et al., 2009; Wen et al., 2018) and cerebral ischemia is often characterized by progressive cognitive deterioration (Clark et al., 2007; Xue et al., 2017). Here we showed that vehicle treated tGCI/R rats exhibited impaired learning and memory abilities in MWM, increased oxidative stress damage and increased paramorphous neurons, apoptosis and degeneration in histopathological examination, which well reproduced our previous findings (Zhang et al., 2018). However, celastrol dose-dependently improved cognitive ability, attenuated oxidative stress and reduced apoptotic neuronal death. Neuronal apoptosis is an important pathophysiological process in cerebral ischemia (Niizuma et al., 2010). Bax/Bcl-2 regulates mitochondrial membrane permeabilization and cerebral ischemia will increase Bax/Bcl-2 ratio thus promote apoptotic neuronal death by facilitating Cyt C release and caspase-3 activation (Xue et al., 2017). Here we indicated that celastrol reversed the increased ratio of Bax/Bcl-2 and inhibited caspase-3 activation after tGCIR. On the other hand, celastrol treatment adjusted the balance between oxidation and antioxidant. Recent studies indicated that celastrol could inhibit neuronal death through modulating mitochondrial autophagy (Lin et al., 2019)and suppressing the induction of mitochondrial reactive oxygen species (Zhang R. et al., 2017). Therefore, it is reasonable to speculate that celastrol may reduce apoptotic neuronal death by inhibiting Bcl-2/Bax dependent caspase-3 activation, thus improve cognitive ability after cerebral ischemia.

Glial cells play important roles in maintaining nervous system homeostasis, they will rapidly activate after brain damage and promote neuroinflammation and oxidative stress, exacerbate delayed neuronal death (Chamorro et al., 2016) and disrupt neuronal processes that are essential for learning and memory (Godinho et al., 2018). Activated microglia/astrocytes promote selective neuronal damage by releasing pro-inflammatory cytokines like TNF-α, IL-1β, and IL-6 and reactive oxygen species (Godinho et al., 2018). Inhibiting microglial/astrocyte activation could attenuate neuroinflammation and oxidative stress thus provide attractive therapeutic strategy (Zhao et al., 2016). Celastrol has been demonstrated to inhibit the activation of microglia and astrocytes (Kim et al., 2009) and we obtained similar results that it partially inhibited tGCI/R induced glial activation and proliferation, lessened inflammatory cytokines and oxidative makers in tGCI/R rats. Thus, we can infer that celastrol may alleviate tGCI/R induced neuroinflammation and oxidative stress via inhibiting glial activation.

Cerebral ischemia may damage cell membranes of neurons and glial-cells, leading to the release of TNF-α, IL-1β, and IL-6. A previous study (Li et al., 2014) demonstrated that levels of pro-inflammatory cytokines were markedly increased both in brain and serum after cerebral ischemia, and that serum pro-inflammatory cytokines level was well correlated with the severity of brain damage. Here we got similar results as a recent study (Jiang et al., 2018) that hippocampal and serum levels of TNF-α, IL-1β, IL-6 were elevated accompanied by a drop in IL-10 level 24 h after middle cerebral artery occlusion in rats. IL-1β could increase p53-upregulated modulators thus promote neuronal apoptosis and structural injury (Zhang et al., 2014; Zhang X. et al., 2017). As potent anti-inflammatory cytokine, IL-10 could attenuate neuroinflammation and promote neurological rehabilitation in spinal cord injury rats (Dietrich et al., 1999) and reduce TNF-α production by regulating both astrocytes and microglia (Jesus et al., 2013). We showed that celastrol could attenuate the increase of pro-inflammatory cytokines and reversed the decrease of anti-inflammatory cytokine in tGCI/R rats.

Oxidative stress could exacerbate brain damage induced by cerebral ischemia (Chen et al., 2011). Enhancement of antioxidant defense system including superoxide dismutase (SOD), glutathione peroxidase (GPx), and catalase (CAT) may be beneficial for neuronal recovery from ischemia-reperfusion injury. Glutathione (GSH) is an important cellular antioxidant and its level indirectly reflects the enzymatic activity of GPx (Xue et al., 2017). Celastrol has been proven to exert antioxidant effects by reducing ROS generation (Yu et al., 2010) and promoting GSH redox cycle (Sravanthi and Rao, 2016). Consistent with our previous study (Zhang et al., 2018), tGCI/R caused evident oxidative stress, whereas celastrol decreased hippocampal MDA level, increased GSH level and enhanced the activity of SOD and CAT in tGCI/R rats. All of the above results suggest that celastrol may exert neuroprotective effects through its anti-inflammatory and antioxidant actions.

NF-κB is a key transcription factor associated with inflammation, oxidative stress and apoptosis. In rat tGCI/R model, antioxidants prevented the phosphorylation and decrease of IκBα in cytoplasm, and reduced NF-κB activity 6 h after reperfusion (Shen et al., 2003). Here we found that celastrol reversed tGCI/R induced phosphorylation and degradation of IκBα, and rescued nuclear translocation of NF-κB subunit p65. Celastrol was previously demonstrated to inhibit the activation of NF-κB pathway, and thereby decrease inflammatory cytokines release (Li et al., 2012). Here we indicated that celastrol mitigated neuroinflammation and oxidative stress as reflected by downregulating pro-inflammatory cytokines like TNF-α, IL-1β, IL-6 and lipid peroxidation product like MDA, whereas anti-inflammatory cytokine IL-10 and anti-oxidative substances GSH, SOD, and CAT were upregulated. As NF-κB has been considered as redox and inflammatory sensitive transcription factor, and its inhibition prevents ROS induced apoptosis (Lee et al., 2003), we may deduce that the neuroprotective effects of celastrol could be attributed to the suppression of neuroinflammation and oxidative stress via inhibiting of NF-κB activation.

HMGB1was originally characterized as nuclear DNA-binding protein, which may release into extracellular space and induce microglial activation and neuroinflammation in post-ischemic brain (Jiang et al., 2012; Tao et al., 2015). HMGB-1 increases under ischemic condition, binding to its receptors like RAGE and TLR-4 and promotes cytokines releasing in a process that involves NF-κB activation (Chen C.B. et al., 2017). HMGB1 was found to increase the expressions of TNF-α and IL-6, which may promote its own release in positive-feedback manner, thus aggravating inflammatory responses (Yang et al., 2016). Here we showed that celastrol lessened tGCI/R induced HMGB1 upregulation and IκBα phosphorylation, and reversed the nuclear translocation of NF-κB. Therefore, we may suggest the neuroprotective effects of celastrol could be largely attributed to the suppression of inflammatory cascades via an HMGB1-dependent NF-κB signaling pathway.

Some limitations exist in the current study. First, initiation of celastrol treatment immediately after tGCI/R may represent a limitation because the time window for effective post-ischemia treatment was not examined. Indeed, ischemic brain injury may occur during diagnostic or surgical procedures and neuroprotective interventions could be initiated promptly. Therefore, the data obtained with celastrol administered immediately after tGCIR do not invalidate its potential clinical efficacy. Second, we did not explore the effects of celastrol on neurogenesis/vasculogenesis and neuroplasticity after tGCI/R. Third, we did not observe the protective effects of continuous celastrol administration after tGCIR. Therefore, the effects of celastrol on neurogenesis/vasculogenesis and neuroplasticity, and the continuous administration effect of celastrol should be the focus of future investigations.

In summary, the present study validated that acute treatment with celastrol may exert anti-inflammatory and anti-oxidant activities thus conferred neuroprotective effects in tGCI/R rats, the mechanisms may involve inhibiting HMGB1 dependent nuclear/cytoplasm translocation of NF-κB thus depressing HMGB1/NF-κB signaling pathway. Our findings provide valuable information that celastrol may be a promising drug candidate for tGCI/R treatment, which may ultimately lead to develop pharmacological therapies for the treatment of patients with stroke.

## Data Availability Statement

All datasets presented in this study are included in the article/supplementary material.

## Ethics Statement

The animal study was reviewed and approved by the Animal Use and Care Committee for Laboratory Animals of Tongji Medical College.

## Author Contributions

BZ, CZ, and XDC conceived and designed the experiments. BZ, QZ, XHC, XW, RS, and GS were responsible for implementing the experiments. BZ and QZ were responsible for data statistics and analysis and wrote the manuscript draft. CZ and XDC supervised the project and revised the manuscript. All authors have read and agreed with the final version of the manuscript.

## Conflict of Interest

The authors declare that the research was conducted in the absence of any commercial or financial relationships that could be construed as a potential conflict of interest.
