# Corrigendum: Neuroprotective Effects of Celastrol on Transient Global Cerebral Ischemia Rats via Regulating HMGB1/NF-κB Signaling Pathway

**DOI:** 10.3389/fnins.2021.637004

**Published:** 2021-07-06

**Authors:** Bo Zhang, Qi Zhong, Xuhui Chen, Xi Wu, Rong Sha, Guizhi Song, Chuanhan Zhang, Xiangdong Chen

**Affiliations:** ^1^Department of Anesthesiology, Union Hospital, Tongji Medical College, Huazhong University of Science and Technology, Wuhan, China; ^2^Department of Anesthesiology, Tongji Hospital, Tongji Medical College, Huazhong University of Science and Technology, Wuhan, China; ^3^Department of Anesthesiology, Zhongnan Hospital, Wuhan University, Wuhan, China; ^4^Department of Ophthalmology, Tongji Hospital, Tongji Medical College, Huazhong University of Science and Technology, Wuhan, China; ^5^Department of Rehabilitation Medicine, Enshi Autonomous Prefecture, Hospital of Traditional Chinese Medicine, Enshi, China; ^6^Department of Quality Inspection, Wuhan Institute of Biological Products, Wuhan, China

**Keywords:** celastrol, neuroinflammation, oxidative stress, neurological deficit, cerebral ischemia reperfusion

In the original article, the image of Bax in Figure 2E was misused in the process of manuscript revision. Figure 3B (Iba-1) was inadvertently copied as Figure 2E (Bax). The corrected Figure 2E appears below.

**Figure 2:**
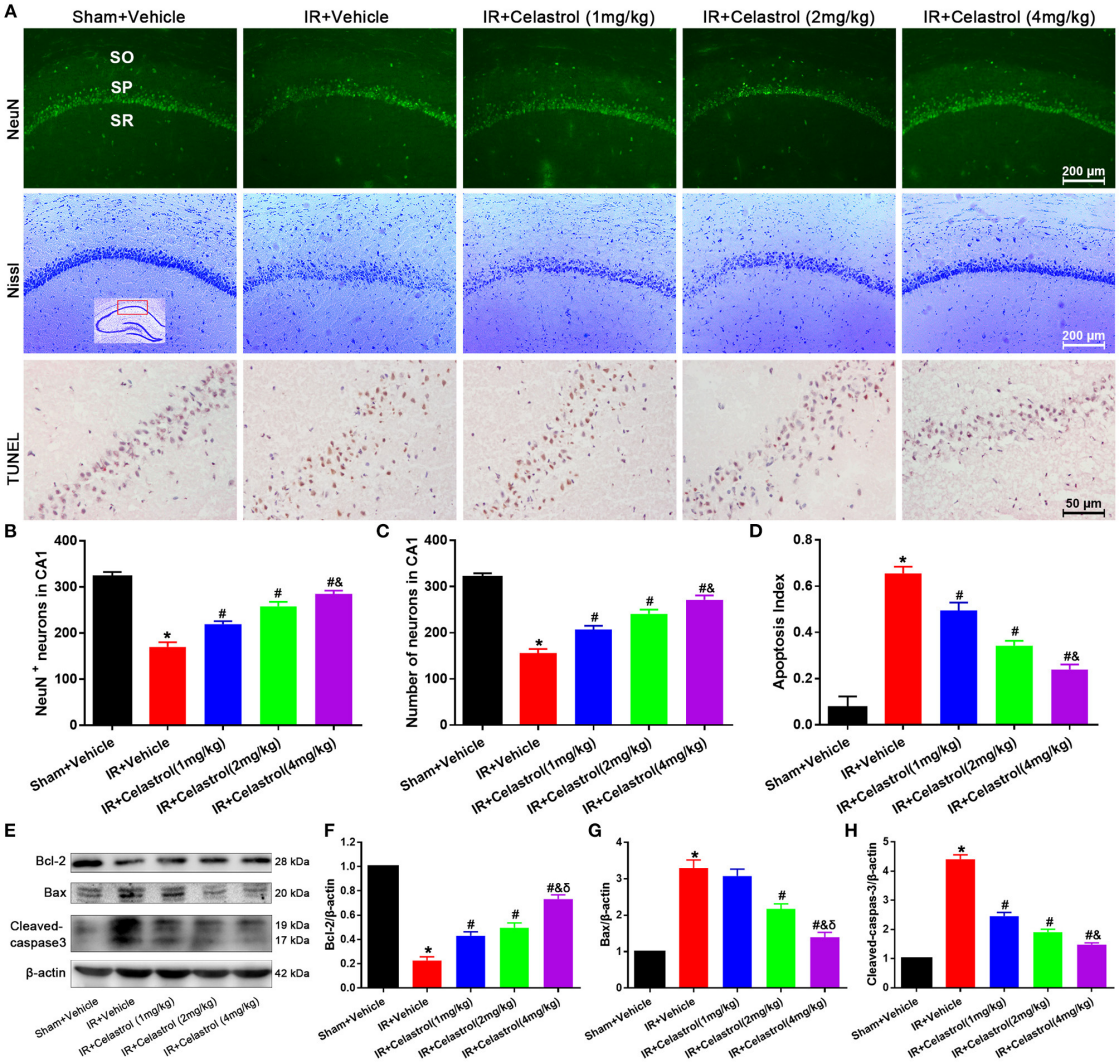
Celastrol inhibits apoptotic hippocampal neuronal death. **(A)** Representative images of NeuN immunofluorescent staining sections (first row), cresyl violet-stained sections (second row) and TUNEL-stained sections (third row) in hippocampal CA1 region of tGCI/R rats, the built-in schematic diagram with red rectangular shows the area that we analyzed. Data were obtained from 4 independent animals, and the results of a typical experiment are exhibited. Scale bar = 200 μm. SO, stratum orients; SP, stratum pyramidal; SR, stratum radium. **(B)** Quantitative analysis of NeuN positive neurons. **(C)** Quantitative analysis of Nissl-positive neurons **(D)** The apoptosis index of hippocampal CA1 neurons. **(E–H)** Western blot analysis of apoptosis-related proteins Bcl-2, Bax and cleaved-caspase-3, β-actin was used as an internal control. The error bars represent mean ± S.E.M (*n* = 4, ^*^P < 0.05 vs. Sham + Vehicle group; ^#^*P* < 0.05 vs. IR+ Vehicle group; ^&^*P* < 0.05 vs. IR + Celastrol (1 mg/kg) group; ^δ^ *P* < 0.05 vs. IR + Celastrol (2 mg/kg) group).

The authors apologize for this error and state that this does not change the scientific conclusions of the article in any way. The original article has been updated.

